# Cellular automata modelling of leukaemic stem cell dynamics in acute myeloid leukaemia: insights into predictive outcomes and targeted therapies

**DOI:** 10.1098/rsos.241202

**Published:** 2025-01-15

**Authors:** Yutaka Saikawa, Toshihiko Komatsuzaki, Nobuaki Nishiyama, Toshihisa Hatta

**Affiliations:** ^1^ Department of Pediatrics, Kanazawa Medical University, Uchinada, Ishikawa 9200293, Japan; ^2^ Faculty of Frontier Engineering, Institute of Science and Engineering, Kanazawa University, Kakuma, Ishikawa 9201192, Japan; ^3^ Graduate School of Natural Science and Technology, Kanazawa University, Kakuma, Ishikawa 9201192, Japan; ^4^ Department of Anatomy, Kanazawa Medical University, Uchinada, Ishikawa 9200293, Japan

**Keywords:** acute myeloid leukaemia, leukaemic stem cells, measurable residual disease, cellular automata

## Abstract

Acute myeloid leukaemia (AML) is a haematologic malignancy with high relapse rates in both adults and children. Leukaemic stem cells (LSCs) are central to leukaemopoiesis, treatment response and relapse and frequently associated with measurable residual disease (MRD). However, the dynamics of LSCs within the AML microenvironment is not fully understood. This study utilized three-dimensional cellular automata (CA) modelling to simulate LSC behaviour and treatment response under induction chemotherapy. Our study revealed: (i) a correlation between LSC persistence post-induction chemotherapy and risk of AML relapse; (ii) MRD negativity based on LSC count may not reliably predict outcomes, supporting clinical evidence that patients with MRD-negative status can still be at risk of relapse; (iii) prolonged persistence of LSCs post-chemotherapy without disruption of normal haematopoiesis, aligning with clinical observations of dormant AML clones; (iv) early LSC dynamics post-induction chemotherapy, characterized by stochastic behaviours and movement velocities, are insufficient predictors of long-term prognosis; and (v) a distinct spatiotemporal organization of LSCs in later phases post-induction chemotherapy is correlated with long-term outcomes. Our modelling results provide a theoretical and clinical framework for AML research, and future clinical data validation could refine the utility of CA modelling for oncological studies.

## 1. Introduction

Acute myeloid leukaemia (AML) is a haematologic malignancy characterized by the clonal expansion of abnormal myeloid progenitor cells that fail to differentiate and function properly, thus disrupting the production of normal blood cells in the bone marrow (BM) [1]. This leads to clinical manifestations such as anaemia, increased risk of infection due to neutropenia and increased risk of bleeding disorders due to thrombocytopenia. AML arises when normal myeloid progenitors or haematopoietic stem cells (HSCs) transform into leukaemic cells (LCs), which include abnormal or incompletely differentiated immature progenitors, or blasts, of the myeloid lineage. This transformation is often driven by genetic mutations that promote uncontrolled growth and inhibit differentiation. AML remains highly resistant to primary chemotherapy, with relapse rates of >50% in adults [2] and 30–40% in children [3], despite recent advances in therapeutic strategies.

A heterogeneous mix of cell populations exists in AML, including a critical subset known as leukaemic stem cells (LSCs), first identified by Lapidot *et al*. [4]. This discovery shifted our understanding of leukaemia, establishing it as a hierarchical disease, with LSCs at the top of this hierarchy. This paradigm shift has highlighted the need for novel treatment strategies aimed at eradicating LSCs to prevent relapse and improve patient outcomes [4,5]. These LSCs exhibit several defining characteristics [5]: (i) self-renewal: LSCs can replicate, generating more LSCs and maintaining the leukaemic population; (ii) differentiation: LSCs possess a limited potential to differentiate into more mature LCs, which constitute the bulk of the leukaemic mass; (iii) treatment resistance: LSCs resist conventional chemotherapies through various mechanisms, contributing to the presence of measurable residual disease (MRD) and frequently leading to relapse in AML; and (iv) the ability to enter quiescence: LSCs often become dormant, evading drugs active for proliferating cells and potentially triggering AML recurrence by escaping initial therapies.

MRD is defined as post-therapy persistence of leukaemic cells, including therapy-resistant leukaemic clones or blasts [2,6]. The assessment of MRD in AML serves multiple purposes. It can be used to: (i) quantify the depth of antileukaemia response, such as determining the log reduction after two cycles of therapy; (ii) detect early signs of relapse through sequential monitoring, allowing for prompt intervention; (iii) guide therapeutic decisions, including the selection of transplant intensity where otherwise equipoise; (iv) select patients for clinical trials, particularly those classified as high risk with unmet medical needs; and (v) act as a surrogate endpoint for overall survival in studies seeking regulatory approval [2,6–9].

The concepts of LSCs and MRD, originating from distinct fields in oncology and haematology, are now closely related in understanding disease persistence, relapse and treatment strategies [2,4,5]. Since LSCs are a primary, but not the only, source of MRD, the presence of MRD after treatment may indicate that LSCs, along with other resistant cell populations, remain active in the body. To achieve deep and lasting remission, treatment strategies should target both LCs and resistant LSCs in MRD [6–9].

Highly sensitive techniques such as multiparameter flow cytometry (MFC), polymerase chain reaction (PCR) and next-generation sequencing (NGS) are utilized to detect MRD [2,6–9]. The definition of clinical MRD negativity varies depending on the sensitivity of the detection method used. For example, the sensitivity of MFC (detecting aberrantly expressed antigens on LCs) ranges from 0.01 to 0.1%; for PCR (detecting gene abnormalities through amplification), it ranges from 0.0001 to 0.01%; and for NGS (identifying gene mutations), it ranges from 0.0001 to 0.001% [10,11]. Although MRD can be detected at very low frequencies, monitoring is challenging due to the lack of LSC-specific markers, which leads to a focus on LCs rather than LSCs. Therefore, it is essential to develop LSC-inclusive MRD detection strategies. Additionally, the variability in LSC behaviour after treatment increases the risk of underestimating MRD and misinterpreting disease eradication. Clinical monitoring of MRD is further complicated by reliance on single-time-point BM analyses that do not reflect the dynamic nature of LC behaviours. To compensate for the limitations of real-time observation, various mathematical models have been developed as supplementary and essential tools for understanding leukaemic pathophysiology, which remains incompletely understood despite these efforts [12–20]. Further refinement of models that explore LSC dynamics is necessary to gain more comprehensive biological insights.

Cellular automata (CA) modelling is a unique computational technique that simulates complex biological systems using simple rules on a discrete lattice [21]. CA modelling can be used to examine emergent behaviours from a spatiotemporal perspective and generate insights into the mechanisms that drive complex systems [22–25]. Since BM haematopoietic cells and cancer cells develop and expand in finite three-dimensional spaces, their behaviours are essentially affected by spatial restrictions, making them particularly well suited for CA model analysis [26–32]. Building on our previous research that employed three-dimensional CA modelling to study normal myelopoiesis [33,34], our current study extended this approach using a novel LSC-centric model of AML designed to visualize and analyse LSC dynamics in AML in the context of post-induction chemotherapy, thereby enhancing our understanding of treatment responses and clinical outcomes.

## 2. Methods

### 2.1. Compartmentalization of normal myelopoiesis

**Figure 1.**
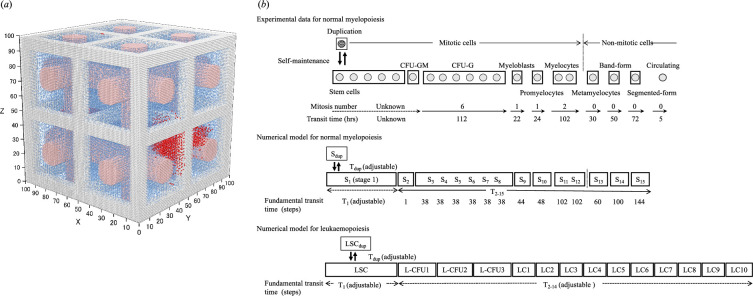
Three-dimensional CA model. (*a*) Three-dimensional distribution of normal myelocytic lineage cells (blue dots) and leukaemic lineage cells (red dots) with simulated structures, trabecular bones (grey dots) and sinusoidal vessels (pink dots), in the BM. (*b*) Compartmentalization of normal myelopoiesis and LSC-driven leukaemopoiesis. CFU-GM, colony-forming unit–granulocyte/macrophage; CFU-G, colony-forming unit–granulocyte; CFU-M, colony-forming unit–macrophage; L-CFU, leukaemic colony-forming unit; LC, leukaemic cell.

The myelopoietic differentiation process was partitioned into 10 compartments, with each cell stage characterized by transit time (*T*), number of mitoses and the fraction of actively proliferating cells, as previously reported [33,34] (see electronic supplementary material, figure S1). For our simulations, stages 1–15 were arbitrarily designated as state variables (figure 1). Fundamental transit time steps (stages 3−15, simulation step = 0.5 h) reflecting cell maturation were determined using transit times (in hours) extracted from published experimental data [35,36]. The stem cell compartment (stage 1) comprised pluripotent stem cells with self-renewal capacity and cells in the early myelocytic lineage stage. Given the uncertainty surrounding early stem cell differentiation and self-renewal, the provided transit times (*T*_1_) and (*T*_dup_) were considered adjustable in the absence of experimental data. Model parameters, including transit time and the number of mitoses for normal myelopoiesis, were sourced directly from the literature or inferred from published experimental data [35,36].

### 2.2. Compartmentalization of LSC-driven leukaemopoiesis

To approximate LSC-driven leukaemopoiesis using CA models, we divided the leukaemic process into 15 compartments, as illustrated in figure 1. These compartments encompassed various subpopulations of LSCs, including those resembling lymphoid lineage progenitors and more mature populations close to colony-forming unit–granulocyte/macrophage (CFU-GM), similar to their normal counterparts [37]. These subpopulations were integrated into the compartments as leukaemic colony-forming units (L-CFUs) 1 to 3, with the subsequent 10 compartments of proliferating LCs arbitrarily designated LC1 to 10. Given the lack of experimental data, all model parameters describing LSC-driven leukaemopoiesis were considered adjustable.

### 2.3. Stem cell division

Both HSCs and LSCs can undergo cell division, resulting in the generation of two, one or no daughter stem cells until they differentiate into CFU-GM or L-CFU1 in a stochastic manner. Stochasticity is characterized by the probabilistic description of stem cell division processes as *p* (producing two daughter stem cells, equivalent to symmetric division), *r* (producing one daughter stem cell, equivalent to asymmetric division) and *q* (producing no daughter stem cells), with the constraint that *p + r + q* = 1. The number of stem cells expands if *p > r +* q, whereas extinction occurs for a finite population if *p < r + q*. A strict steady state is maintained when *r* = 1, whereas a stationary state is possible if *r* < 1 and *p = q*.

### 2.4. Development of CA models for LSC-driven AML with chemotherapy

The normal myelopoietic CA models were established within a three-dimensional space consisting of 1 000 000 cubic units organized in a 100 × 100 × 100 lattice. To simulate an infinite structure and accurately depict dynamic processes within continuous space, we employed periodic boundary conditions, treating the lattice as a continuous ring in each dimension. This configuration seamlessly connected opposite ends of the lattice. Each cube in the lattice represented the size of a single biological cell. Additionally, the analytical space included structural objects resembling sinusoidal vessels and trabecular bones, which accounted for 33% of the BM cavity (figure 1).

To represent cell distributions in space, two types of state variables were assigned to each unit area. First, a set of cell states was defined to differentiate cells based on their location and proliferation stage. This set comprised a total of 32 cell states, which included cases of cell absence, vessel parts and bone fragments, based on assumptions related to the normal myelopoietic and leukemopoietic processes (figure 1, numerical models). Second, cell age was defined as the second state variable, with incrementation at every simulation step to represent cell maturation until reaching the respective transit time for the next stage.

To develop CA models of LSC-driven AML, we introduced LSCs into our normal steady-state myelopoietic models to initiate leukaemopoiesis. Simulation runs were conducted using different numbers of LSCs (e.g. 1 to ≥10 for a shorter period of AML development), with each LSC randomly assigned to distinct spatial locations and ages at the start of every simulation run.

The modelling for AML treatment incorporated the mechanisms of action of common antineoplastic drugs used to treat AML, which target mitosis to induce cell death. These drugs affect both LCs, including LSCs and normal mitotic myelopoietic cells [38]. In our AML treatment models, cells in mitotic compartments (stages 1–12 of normal myelopoiesis, LSC-LC10 of leukaemopoiesis and self-renewal of HSCs and LSCs) were eliminated from the model at a constant disappearance rate during drug exposure periods. For treatment simulations, we adopted the AML-05 treatment protocol designed for paediatric patients with AML, approved by the Japanese Pediatric Leukemia/Lymphoma Study [39] (electronic supplementary material, figure S2). A representative simulation model examined a regimen consisting of 12 consecutive days of treatment followed by a three-week interval, repeated over five cycles. This regimen was designated as ‘induction chemotherapy: 12 consecutive days × 3-week interval × 5 cycles’, as illustrated in electronic supplementary material, figure S2. The disappearance rates for the simulations are shown in electronic supplementary material, table S3. The above-mentioned scheme was implemented in the ‘Biosim’ simulation code [40].

### 2.5. Description of the local neighbour rules for CA models

Cellular state variables were updated synchronously based on local neighbour rules at each calculation step. These rules governed cell movements and transitions to different stages, depending on the unit element’s state, and microenvironmental effects from neighbouring elements. Essentially, cells could move to any of the 26 randomly selected neighbouring sites at each time step. To prevent collisions with other cells at neighbouring sites, conflicting movement directions were resolved using the following process: each cell was mapped, and before moving, a direction was randomly chosen from nearby vacant sites. If conflict occurred at a destination, one cell was randomly selected to move, whereas the other cells remained in their current locations. As a result, cellular behaviours can be described as follows: cell proliferation and division were limited, with cells remaining in their current state if no adjacent vacancy was available. Consequently, cells could exceed their transit time steps. A flowchart detailing the simulation process is provided in electronic supplementary material, figure S3.

### 2.6. Assumptions in the models

— Both HSCs and LSCs behave independently.
— All events related to stem cells, encompassing self-renewal and differentiation, occur stochastically.
— HSCs are deterministically programmed to differentiate towards neutrophils.
— All clones originating from a single HSC contribute equally to myelopoiesis; therefore, specific interactions with cells of different lineages (e.g. lymphocytic lineage cells, erythrocytic lineage cells, megakaryocytic lineage cells) were not considered.
— The duration of apoptotic events was assumed to be significantly longer, set at approximately 1000 times the total transit time for normal myelopoiesis and 400 times for leukaemopoiesis.
— Quiescence of stem cells (HSCs and LSCs in a reversible G0 cell cycle state) was not considered.
— Functions of the BM cavity, in which the cells proliferate and interact with a vast vascular network and non-haematopoietic cells that form the microenvironment and niches, were not assumed.

### 2.7. Construction of cell movement trajectories

Each cell at time *t* retained information regarding its coordinates at time *t* − 1, one time step before. Consequently, cell trajectories were tracked during the simulation and reconstructed using our custom software, ‘CellTrajectory’ [40]. The period during which each cell remained at the same position in space was defined as the dwell time, and the length of the dwell time varied with changes in the cell’s microenvironment. To demonstrate the continuity of cell movement trajectories across the initial periodic boundary line, alternative boundary lines were introduced to maintain the continuous lattice structure. This adjustment ensured the preservation of spatial relationships and enabled the simulation of dynamic processes across the entire lattice. To visualize the distribution of cells with dwell time, spheres of various sizes corresponding to the length of the dwell time were displayed on the trajectory lines. Three-dimensional graphical maps for visualizing cellular trajectories were created using Origin 2022 software.

### 2.8. Centroid vectors of cell movement trajectories

The centroid of a cell movement path was defined as the arithmetic mean position in three-dimensional space, representing the location where the cell was assumed to remain within a given observation time. To calculate the dwell time-weighted coordinate value for a cell, the following equations were utilized at intervals of every 200 calculation steps, with the results then averaged over 1000 steps:


(2.1) $$\begin{array}{l} X_{cent}=\sum_{i=1}^{n}T_{d}^{i}X_{i}/\sum_{i=1}^{n}T_{d}^{i},\\ Y_{cent}=\sum_{i=1}^{n}T_{d}^{i}Y_{i}/\sum_{i=1}^{n}T_{d}^{i},\\ Z_{cent}=\sum_{i=1}^{n}T_{d}^{i}Z_{i}/\sum_{i=1}^{n}T_{d}^{i}. \end{array}$$

In these equations, ($X_{\text{cent}}$, $Y_{\text{cent}}$, $Z_{\text{cent}}$), ($X_{i}$, $Y_{i}$, $Z_{i}$) and $T_{d}^{i}$ represent the centroid position, the cell position and the dwell time at every 200 time steps, respectively. Additionally, two adjacent centroid points were linked to form a centroid vector.

### 2.9. Histograms of cellular dwell times

Histograms were constructed to visualize the distribution of relative dwell time frequencies for all cells present at a given time. To assess the disparity in dwell time distributions between the cure and relapse models, the cosine distance was utilized as a pairwise distance metric [41]. For each bin in the histogram representing dwell time distribution, a pair of vectors was formed, *X*_*i*_ and *Y*_*i*_, containing relative frequencies. The cosine distance, denoted as *D*_cos_, was then calculated by subtracting the cosine similarity from 1:


(2.2) $$D_{cos}\left(X,Y\right)=1-\frac{\sum_{i=1}^{n}X_{i}Y_{i}}{\sqrt{\sum_{i=1}^{n}X_{i}^{2}\sum_{i=1}^{n}Y_{i}^{2}}}.$$

The parameter *n* denotes the number of bins in the histogram. A *D*_cos_ value of 0 indicates that the histograms are identical. This measure enabled the quantification of similarities or dissimilarities between the dwell time distributions in the two models. *D*_cos_ was computed using the pdist2 function in Matlab 2022.

### 2.10. Construction of two-dimensional contour diagrams

Two-dimensional contour diagrams were generated to visualize the spatial distribution of neighbouring cells derived from stem cells, each affected by its unique microenvironmental condition within the analytical three-dimensional space. These contour diagrams were generated using either Origin 2022 or Matlab 2022. To maintain a consistent representation of dwell time levels within the analytical three-dimensional space, alternative boundary lines were not introduced during the generation of the contour diagrams. Contour lines, interpolated from the trajectory simulation data, represent areas of equal dwell time values. Each contour line corresponds to a specific level of dwell time, providing a visual representation of the spatial variation in dwell time across the analytical three-dimensional space. To enhance visualization, the contour diagrams were colour-coded using Color Universal Design principles for colour mapping.

## 3. Results

### 3.1. Establishment of CA models for LSC-driven AML with chemotherapy

We previously described the creation of three-dimensional CA models for normal myelopoiesis based on the compartmental architecture of haematopoiesis (see electronic supplementary material, figure S1). These models begin with the simulated proliferation of a single HSC and lead to one of three possible outcomes: steady state, extinction or oscillation, depending on the selected parameter values. Key parameters include the rate of self-renewal (*T*_dup_), HSC differentiation (*T*_1_) and myelocytic differentiation (*T*_2–15_) (figure 1). We achieved a homeostatic steady state in myelopoiesis under specific conditions, as shown in electronic supplementary material, table S1. For further analyses, the following parameter values were chosen to avoid extinction or oscillation: *T*_dup_ = 600, *T*_1_ = 1200 and *T*_2–15_ = 829. These parameters resulted in a total cell count of approximately 100 000 and an average of 200 HSCs per analytical space at steady state, maintaining the HSCs at an average frequency of 0.2% within the models.

For models simulating AML driven by LSCs, we introduced a varying number of LSC clones, each with a unique cellular state, into the established steady-state myelopoietic models. During these simulations, the LSC clones produced clonal LCs that competed for space in their microenvironment with both normal myelocytic lineage cells and other LSC clones. AML was considered established when LCs comprised ≥20% of the BM, as achieved using the parameters listed in electronic supplementary material, table S2.

**Figure 2.**
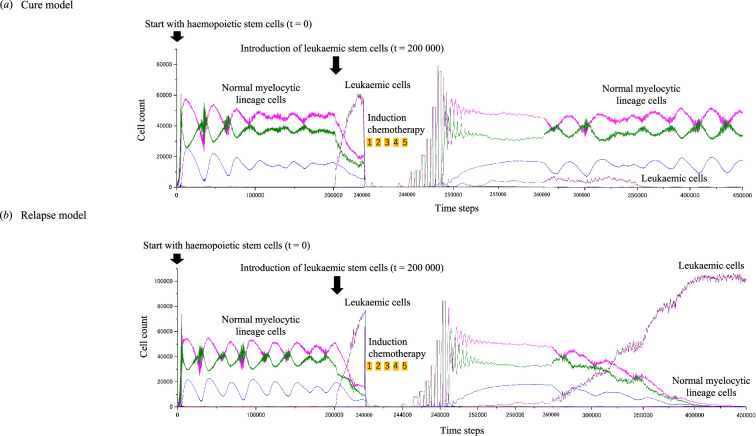
Cell dynamics in the CA models for LSC-driven AML with chemotherapy. Varying numbers of LSC clones were introduced into the model at the steady state of normal myelopoiesis. Following the establishment of AML conditions, treatment was initiated by eliminating mitotic cells at a constant rate, as detailed in electronic supplementary material, table S3. Continued simulations up to *t* = 450 000 (equivalent to a simulated 26-year period) resulted in two distinct outcomes: (*a*) cure or (*b*) relapse.

Treatment simulations followed an ‘induction chemotherapy: 12 consecutive days × 3-week interval × 5 cycles’ regimen and were initiated by eliminating mitotic cells at a constant rate during the drug exposure periods, as detailed in electronic supplementary material, table S3. Simulations continued until the time (*t*) step reached 450 000, equivalent to a simulated 26-year period, with each time step representing a 0.5 h increment. Two outcomes were observed: cure (disappearance of LSCs) or relapse (return of LSCs). Figure 2 illustrates the cell dynamics through a series of simulation events, including introduction of LSCs and AML treatment within the CA models of LSC-driven AML treatment. Notably, to achieve a cure with chemotherapy, leukaemopoiesis required approximately three times the number of time steps than were needed for normal myelopoiesis, as shown in electronic supplementary material, table S2. Without this extended time frame, all simulations resulted in relapse.

### 3.2. Prognostic relationship between residual stem cells in CA models for LSC-driven AML with chemotherapy

**Figure 3.**
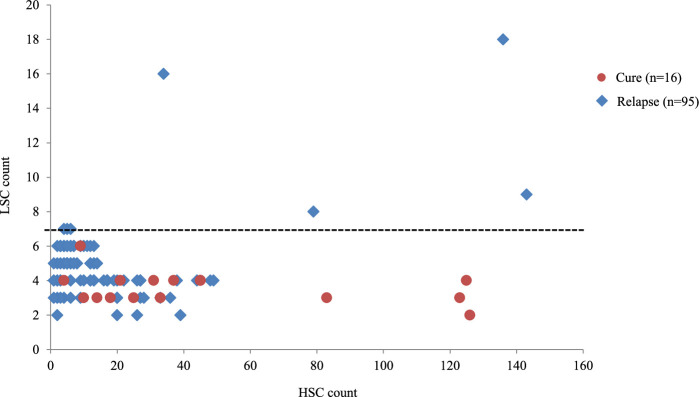
Prognostic relationship between residual stem cells post-induction chemotherapy. Scatter plot illustrating the prognostic relationship between the number of residual HSCs and LSCs following chemotherapy. Simulations with varying rates of mitotic cell elimination produced differing proportions of residual HSCs and LSCs. Cure outcomes are represented by red circles, relapse by blue diamonds and the dashed line indicates the threshold of seven residual LSC clones. HSC, haemopoietic stem cell; LSC, leukaemic stem cell.

We initially examined the prognostic relationship between the number of residual HSCs and LSCs in our CA models. Varying the disappearance rate of different mitotic cells during treatment simulations led to differing proportions of residual HSCs and LSCs (figure 3). Simulations with ≥7 residual LSC clones consistently resulted in relapse, regardless of the residual HSC count. Moreover, the presence of ≥10 residual LSC clones was associated with MRD positivity, clinically defined as >0.01% LCs in the BM [10,11]. This finding was consistent with clinical data indicating that MRD positivity at the first remission increases relapse risk [10]. In contrast, simulations with ≤6 LSC clones, which reflect MRD negativity (≤0.01% LCs), resulted in mixed outcomes of either cure or relapse. For conditions of MRD negativity, the correlation between the number of HSCs and LSCs for cure was *r* = 0.014 and *r* = 0.424 for relapse. These low correlations suggest that the number of residual LSCs or HSCs alone is not a reliable outcome predictor. These data appear to support clinical findings that MRD-negative patients can still experience relapse [7–10].

### 3.3. Relative frequencies of outcomes

To determine the relative frequencies of different outcomes (cure or relapse) under MRD-negative conditions, we varied the number of residual HSCs and LSCs in the simulations. Each simulation was independently repeated with an identical initial cellular status for each residual HSC and LSC at the end of chemotherapy, which included location in space, cell differentiation stage and age (electronic supplementary material, table S2). Table 1 shows that the relapse frequency increases with an increase in the number of LSCs, indicating that even a single remaining LSC clone can potentially initiate AML relapse in our models. For further analysis, we employed conditions from simulation file number 2, which started with 75 HSCs and 2 LSCs and resulted in a relapse rate of 50%.

**Table 1.** Relative frequencies of outcomes. HSC, haemopoietic stem cell; LSC, leukaemic stem cell.

simulation file number	numbers of residual stem cells		numbers of simulation run	numbers of outcomes		relative frequencies	
	HSC	LSC		cure	relapse	cure	relapse
1	74	1	29	25	4	0.86	0.14
2	75	2	40	20	20	0.50	0.50
3	125	3	40	14	26	0.35	0.65

### 3.4. Cell dynamics in CA models for LSC-driven AML after chemotherapy

**Figure 4.**
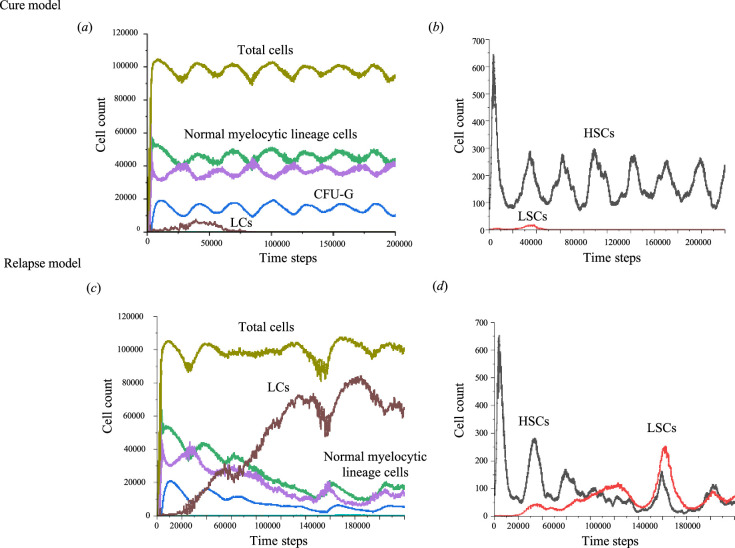
Dynamics of cell populations in CA models for LSC-driven AML post-induction chemotherapy. Representative cell dynamics within the CA models for LSC-driven AML from immediately after chemotherapy (*t* = 0) to *t* = 200 000 in (*a,b*) the cure model and (*c,d*) the relapse model. In the cure model, residual HSCs exhibited cyclic oscillations in proliferation. In the relapse model, these cyclic oscillations of HSCs were progressively disrupted, leading to a decline in normal myelocytic lineage cells. HSCs, haemopoietic stem cells; CFU-G, colony-forming unit–granulocyte; LSCs, leukaemic stem cells; LCs, leukaemic cells.

To explore the presence of determinant events for outcomes within our CA models, we initially analysed the post-chemotherapy cell dynamics of HSCs and LSCs in both the cure and relapse models. These simulations followed the parameters set in simulation file number 2 from table 1, which demonstrated a 50% relapse rate. Figure 4 shows the representative results of these simulations. Following chemotherapy, a small number of HSCs (75 clones) and LSCs (2 clones) survived in both models. In the cure model, the residual HSC population demonstrated oscillatory behaviour, eventually stabilizing into cyclic proliferation patterns, whereas the two LSCs were eliminated at *t* = 10 822 and *t* = 48 139. Notably, LSCs could persist for a time equivalent to up to approximately 3 years (*t* = 48 139) post-chemotherapy without adversely affecting normal myelopoiesis, with frequencies reaching a maximum of 0.018% for LSCs at *t* = 38 273 and 7.3% for LCs at *t* = 39 087. Conversely, in the relapse models, an increase in the number of LSCs disrupted the HSC oscillations, resulting in diminished production of normal cells. Of the two LSCs, one continued to proliferate, whereas the other was lost at *t* = 5359.

### 3.5. Cell movement trajectories of residual LSCs during the early phase after chemotherapy

**Figure 5.**
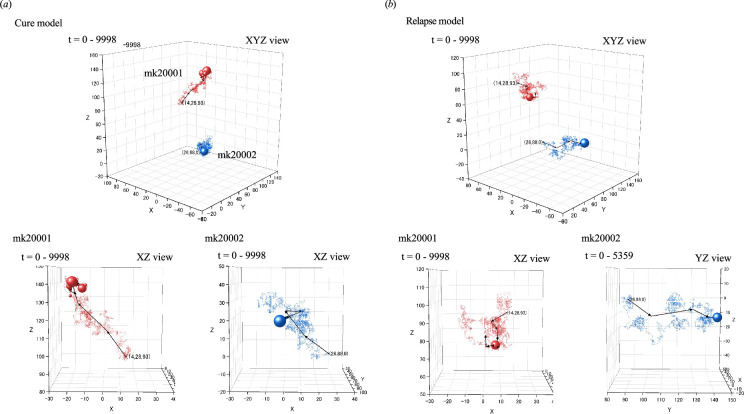
Initial-phase LSC movement trajectories post-induction chemotherapy. The trajectories of LSCs from *t* = 0 to 9998 are represented by centroid vectors. The size of each sphere plotted along the trajectories corresponds to the dwell time of the LSCs at that location. Coordinates labelled in the panels indicate the starting points of LSCs. In the cure model (*a*), LSC mk20001 and mk20002 disappeared at *t* = 48 139 and *t* = 10 822, respectively. In the relapse model (*b*), mk20002 disappeared at *t* = 5359. The tracking simulation runs started at the end of chemotherapy (*t* = 0).

To elucidate the mechanisms by which LSCs disrupt the cyclic oscillations of HSCs, we tracked the paths of these cells immediately after chemotherapy. We then created three-dimensional graphical representations of the movement trajectories to facilitate analysis. These trajectory analyses were based on simulations with a 50% relapse rate, as described in simulation file number 2 from table 1. As shown in figure 5, the two LSCs (designated mk20001 and mk20002) exhibited a stochastic movement pattern during the initial phase (*t* = 0–9998) in both the cure and relapse models. For example, mk20001 ascended from its starting point in the cure model but descended in the relapse model, whereas mk20002 ascended in the cure model and traversed horizontally in the relapse model. The trajectory lines are marked with spheres of varying sizes in the figure as an indication of LSC dwell time, defined as the amount of time a cell remains at a particular location in space. These spheres are accumulated primarily towards the latter part of the observation period. In the cure model, mk20001 and mk20002 disappeared at *t* = 48 139 and 10 822, respectively. In contrast, in the relapse model, mk20001 continued to proliferate, whereas mk20002 disappeared at *t* = 5359. The eradication of LSCs corresponds to their transition from an LSC state to an L-CFU-1, which is considered their functional disappearance.

### 3.6. Three-dimensional vector diagrams of stem cell movement in the relapse model during the early phase after chemotherapy

**Figure 6.**
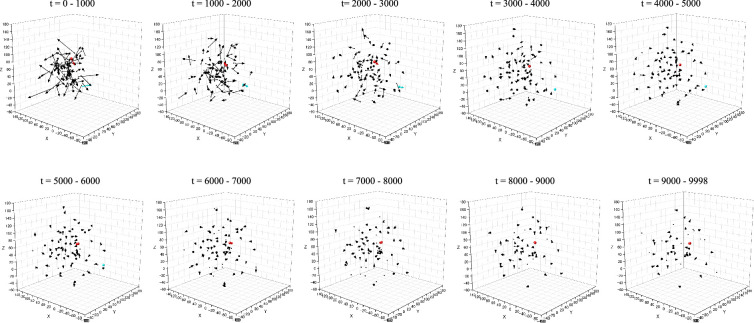
Three-dimensional vector diagrams of stem cell movements in the relapse model after chemotherapy, generated from trajectory data (simulation file number 2 in table 1) at 1000 time-step intervals. Each trajectory is shown as a vector connecting its starting and ending points. Black, red and blue arrows represent the vectors of HSCs, mk20001 and mk20002, respectively.

Stem cell movement was comprehensively analysed by generating three-dimensional vector diagrams from trajectory data, extracted from simulation file number 2 in table 1, at every 1000 time-step interval. Figure 6 illustrates the stochastic movement patterns of both HSCs and LSCs within the relapse model, showing a pattern of gradual dispersal characterized by decreasing movement velocity. Notably, in the initial period from *t* = 0 to *t* = 9998, a substantial reduction in the stem cell population was observed, with 33 of 75 HSCs and 1 of 2 LSCs disappearing. These findings, considered along with the detailed movement trajectories of LSCs shown in figure 5, highlight the profound impact of microenvironmental factors on stem cell dynamics and cellular differentiation processes in our models.

### 3.7. Histograms of cell dwell times during the early phase after chemotherapy

**Figure 7.**
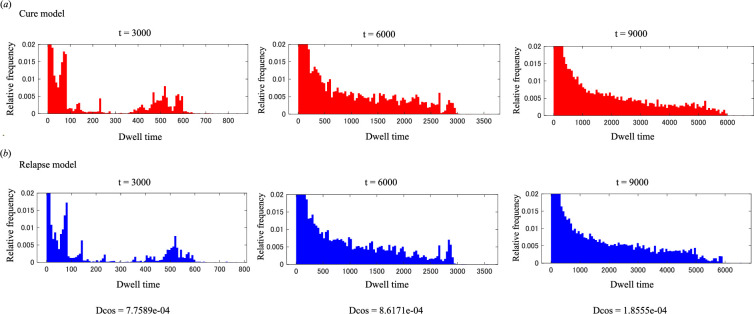
Histograms of cell dwell times in the early phase post-induction chemotherapy. Histograms represent the relative frequency distributions of cell dwell times for all cells in the cure model (*a*) and relapse model (*b*). The number of bins was 100. The cosine distance (*D*_cos_) was calculated to quantify the dissimilarity between the dwell time distributions in the two models, with a *D*_cos_ value of 0 indicating identical histograms.

To distinguish the behaviours of neighbouring cells in the cure and relapse models, dwell times for all cells were compared at distributions of *t* = 3000, 6000 and 9000 using trajectory data from simulation file number 2 in table 1. Histograms were constructed, and cosine distance (*D*_cos_) values were calculated to quantify the dissimilarity between the two models [41]. As illustrated in figure 7, the computed *D*_cos_ values at each time point were close to zero with 100 bins and showed similar values across different binning schemes, ranging from 10 to 5000 bins. These findings suggest that there was virtually no discernible difference in the distribution of relative frequencies between the cure and relapse models at these time points. Furthermore, these observations suggest that microenvironmental conditions, as reflected by the cellular dwell time frequency during the initial phase post-chemotherapy, were comparable between the two models. Consequently, precise prediction of outcomes in the early post-chemotherapy phase may be challenging, underscoring the need for further investigation to identify factors that affect these outcomes.

### 3.8. Distribution of LSC-derived cells in CA models for LSC-driven AML during the later phases after chemotherapy

**Figure 8.**
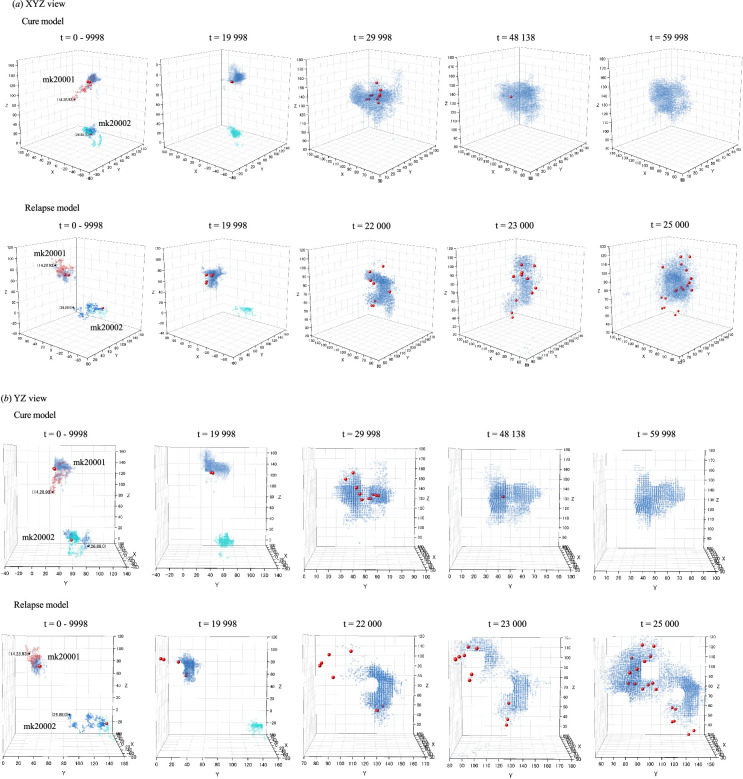
Spatiotemporal distributions of LSC clones and LSC-derived LCs in the later stages post-induction chemotherapy. (*a*) XYZ view, (*b*) YZ view. Blue dots represent LCs derived from mk20001, sky-blue dots indicate LCs derived from mk20002 and red spheres indicate the LSC clones. The trajectories of LSCs from *t* = 0 to 9998 and the distributions of LCs at *t* = 9998 are overlaid for reference.

Figure 8 illustrates the spatiotemporal distributions of LSC clones and LSC-derived LCs following induction chemotherapy in the cure and relapse models, using trajectory data from simulation file number 2 in table 1. In the cure model, the mk20001 clones concentrated within a specific region of the LSC-derived LC distribution at *t* = 29 998. Subsequently, the number of mk20001 clones decreased, eventually disappearing entirely by *t* = 48 139. In contrast, in the relapse model, the mk20001 clones dispersed beyond the initial LC distribution. The mk20002 clones in both the cure and relapse models are not shown in the figures due to their disappearance at *t* = 10 822 and *t* = 5359, respectively. These distinct patterns of cellular behaviour were strongly associated with outcomes in our study, prompting us to undertake additional simulations to explore the effect of the microenvironment on LSC dynamics.

### 3.9. Two-dimensional contour diagrams of the spatial distribution of stem cell-derived neighbour cells during the early phase after chemotherapy

**Figure 9.**
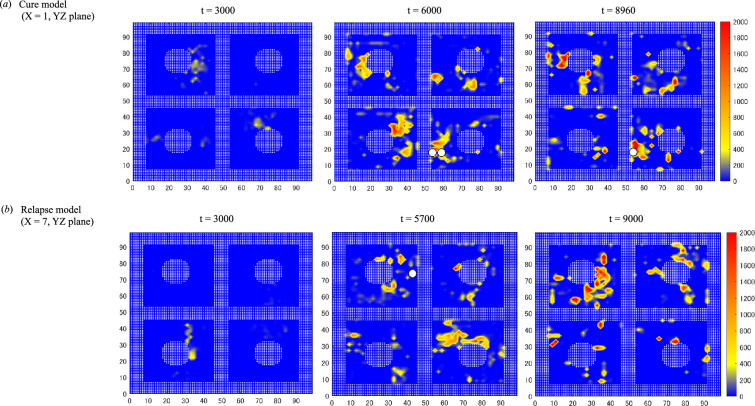
Contour diagrams of cell dwell time distributions post-induction chemotherapy. These two-dimensional contour diagrams compare dwell time distributions in the cure model (*a*) and relapse model (*b*) during the initial post-chemotherapy phase. (*a*) A cross-sectional view at *X* = 1 on the *YZ* plane for the cure model and (*b*) a cross-sectional view at *X* = 7 on the *YZ* plane for the relapse model. LSCs are marked as white circles with black borders. Filled circles and square bars formed by white dots depict vessels and trabecular bones in the BM, respectively. The colour bar indicates cell movement speed, with the gradient from red to blue representing increasing speed. Red suggests slower moving cells with longer dwell times, whereas blue signifies faster moving cells or unoccupied regions.

We examined the spatial distribution of stem cell-derived neighbour cells (normal and leukaemic cells) in the early phase post-chemotherapy using two-dimensional contour diagrams, as shown in figure 9. These diagrams, based on dwell times for all cells, reveal the spatial relationship between LSCs and adjacent stem cell-derived cells using data from trajectory simulations found in simulation file number 2 in table 1. The diagrams show regions of slower cell movement using contour lines that transition from yellow to red to reflect the gradation in motility. During the initial phase (*t* = 0–9000), no visually discernible differences were observed in terms of the distribution of these areas between the cure and relapse models. This observation was further supported by the results of the quantitative histogram analyses shown in figure 7.

### 3.10. Two-dimensional contour diagrams of the spatial distribution of stem cell-derived neighbour cells in the later phases after chemotherapy

**Figure 10.**
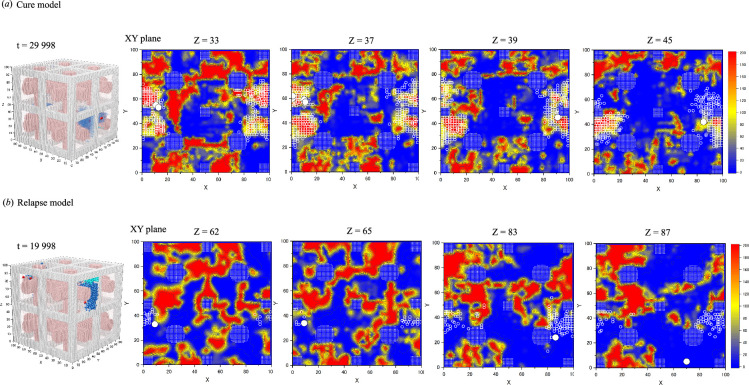
Contour diagrams of cell dwell time distributions at a later stage after chemotherapy. (*a*) The cure model with cross-sectional views at *Z* = 33, 37, 39 and 45 along the *XY* planes at *t* = 29 998, whereas (*b*) illustrates the relapse model at *Z* = 62, 65, 83 and 87 along the *XY* planes at *t* = 19 998. LSCs are depicted as white filled circles with black borders, and LCs are depicted as white open circles. Filled circles and squares formed by white dots show vessels and trabecular bones in the BM, respectively. The colour scheme follows that of figure 9 for consistency. Adjacent to each panel are three-dimensional *XYZ* views at the corresponding times, showing structural objects: grey bars for trabecular bones, pink cylinders for vessels, red dots for mk20001 clones, blue dots for mk20001-clone-derived LCs and sky-blue dots for mk20002-clone-derived LCs. The mk20002 clones are omitted from both models in the figure due to their disappearance at *t* = 10 822 and *t* = 5359, respectively.

As shown in figure 10, the cure model diagrams show clusters of LSCs surrounded by LCs at *t* = 29 998, predominantly in areas indicative of slower cell movement. This pattern suggests a decrease in cell motility, potentially due to microenvironmental factors, leading to the disappearance of LSCs and a successful cure outcome as shown in figure 8. In contrast, the relapse model showed LSCs outside the regions of LC aggregation at *t* = 19 998, with both cell types exhibiting more rapid movement. This increased motility suggests that LSCs and LCs were unhindered, allowing for their proliferation and leading to a relapse outcome, as shown in figure 8.

## 4. Discussion

Our current study focused on the dynamics of LSCs, which is a promising, yet not fully understood, research area. We employed three-dimensional CA models to gain new insights into LSC-driven AML within the context of chemotherapy. These models provide a detailed representation of cell dynamics over an equivalent of two decades, tracing the transition from normal myelopoiesis to the onset of AML, followed by chemotherapy intervention and subsequent outcomes. Key insights include the following aspects.

**A correlation between residual LSC clones and relapse risk.** Our models indicate that a direct correlation exists between the number of residual LSC clones post-treatment and AML relapse risk. Specifically, the presence of ≥10 residual LSC clones is associated with MRD positivity and increased relapse risk. This underscores the clinical importance of MRD assessments in predicting patient outcomes [2,6–9].

**Uncertainty in MRD negativity.** In our models, conditions classified as MRD negative (≤6 residual LSC clones) did not guarantee reliable outcome predictions based solely on LSC or HSC counts. This underscores the unpredictable nature of cell dynamics during the early phase of myelopoietic reconstitution and supports the hypothesis that AML patients, even those achieving MRD negativity after chemotherapy, face a potential relapse risk [7–11].

**Long-term LSC persistence post-chemotherapy.** Our cure model indicated that LSCs may persist for a period equivalent to up to 3 years after chemotherapy without impeding normal myelopoiesis, in contrast to their disruptive role in the relapse model. This result aligns with clinical observations in which AML clones can be detected in patients in remission for extended periods (ranging from 1 to 12.5 years, with a median of 3.75 years) [42]. Investigating this persistence of benign behaviour of LSCs after chemotherapy could inform the development of targeted therapies, such as enhancing microenvironmental factors, to eradicate LSC malignancy without impairing normal haematopoiesis.

**Challenges in predicting outcomes based on early-phase LSC dynamics.** Both models demonstrated similar stochastic behaviours and cell movement velocities among LSCs in the initial phase, suggesting these early dynamics are poor predictors of long-term outcomes. These results emphasize the complexity and unpredictability inherent in MRD negativity assessments [8].

**Later-phase LSC distribution patterns and long-term outcomes.** Our models identified distinct outcome-associated LSC spatial distributions and mobility patterns that appear purposeful in nature. In the cure model, LSCs showed limited mobility and cluster localization, promoting their elimination. Conversely, in the relapse model, LSCs demonstrated increased mobility and peripheral dispersion, which facilitated proliferation. These distinct spatiotemporal distributions and mobility patterns are indicative of emergent properties resulting from the complex interplay of microenvironmental factors affecting cell dynamics. These dynamics, including cell–cell interactions and cell–extracellular matrix interactions, highlight the critical role of the microenvironment in influencing cell behaviours and long-term outcomes.

Our findings underscore the value of three-dimensional CA modelling in elucidating LSC dynamics and offer novel insights that could guide the development of new AML therapies and prognostic tools. However, this modelling study focused specifically on the dynamics of LSCs in relation to AML, MRD and relapse. It is important to acknowledge that although MRD and AML relapse are complex and multifactorial topics, this approach does not fully capture the roles of all leukaemic progenitors and blasts, which would also be crucial in order to obtain a comprehensive understanding. Additionally, potential oversimplification of complex systems, variability in results and the need for a thorough *in vivo* validation should be considered. Future clinical data correlation will further validate and refinethe applicability of CA models in cancer research, establishing their importance in advancing our understanding of AML.

## Data Availability

The underlying codes for this study (‘Biosim’ [40] and ‘CellTrajectory’ [43]) are available at the Dryad data platform. Supplementary material is available at online [44].
